# Sphincter-induced dynamic obstruction in a synthetic intersphincteric anal fistula model: a proof-of-concept fluid–structure interaction study

**DOI:** 10.3389/fmed.2026.1865558

**Published:** 2026-07-10

**Authors:** Qiuxiang Yu

**Affiliations:** Department of Proctology, China-Japan Friendship Hospital, Beijing, China

**Keywords:** anal fistula, biomechanics, dynamic obstruction, fluid–structure interaction, ineffective drainage

## Abstract

**Introduction:**

Perianal abscess often progresses to anal fistula despite timely drainage, and recurrence remains common after sphincter-preserving surgery. Although inadequate drainage is a key factor, existing explanations focus mainly on anatomy, while the role of anal sphincter activity is unclear. This study examined whether sphincter contraction impairs intraluminal flow and contributes to drainage failure.

**Methods:**

A subject-specific fluid–structure interaction model was constructed from high-resolution pelvic MRI. A three-dimensional finite element model of the anal sphincter complex with a synthetic intersphincteric fistula tract was reconstructed. Time-dependent physiological loading simulated sphincter contraction, and bidirectional analyses were performed. Intraluminal velocity fields were quantified using an image-based inverse mapping approach to characterize low-velocity retention zones.

**Results:**

Sphincter contraction induced extensive low-velocity regions despite anatomical patency, with a progressive increase during mid-loading. Connectivity analysis revealed an aggregation–retention–fragmentation cycle, and multi-threshold sensitivity analysis supported the robustness of low-velocity region identification.

**Conclusion:**

In this healthy-volunteer-derived, synthetic intersphincteric fistula model, sphincter contraction generated low-velocity retention zones despite preserved anatomical patency. These findings support a plausible biomechanical hypothesis for ineffective fistula drainage, but should be interpreted as qualitative proof-of-concept evidence pending validation in patient-specific native fistula models and clinical studies.

## Introduction

Perianal abscess and anal fistula are widely regarded as different manifestations of the same cryptoglandular disease process and remain among the most common disorders encountered in colorectal practice. Despite timely incision and drainage of perianal abscesses, a considerable proportion of patients subsequently develop chronic anal fistula, and treatment failure or recurrence remains a substantial problem, particularly in high or complex fistulas, even in the era of sphincter-preserving procedures (1–3). These observations suggest that anatomical patency of a fistula tract alone may not be sufficient to ensure effective drainage and durable resolution.

Adequate drainage of infected intersphincteric or fistulous spaces is a fundamental principle in the management of anorectal sepsis (1). Accordingly, most current surgical strategies are designed to establish, preserve, or optimize anatomical patency of the fistula tract. However, this anatomy-centered framework does not fully explain a common clinical finding: some fistula tracts appear patent on imaging or at surgery, yet drainage remains inefficient or persistent discharge continues. This discrepancy raises the possibility that factors beyond static structural patency may influence fluid transport within the tract.

From a biomechanical standpoint, the anal sphincter complex is an active pressure-generating system. The internal anal sphincter contributes most of the resting anal pressure, while the external anal sphincter and associated pelvic floor musculature further modulate local mechanical loading on surrounding tissues (4). A fistula tract embedded within or adjacent to this dynamically contracting muscular environment may therefore undergo time-dependent deformation, transient narrowing, and compliance changes during sphincter activity. In other compliant conduits, periodic external compression can generate complex flow behavior through fluid–structure interaction, including flow reversal, recirculation, and low-velocity regions, even when the lumen remains anatomically patent (5–7). These phenomena are conceptually related to valveless impedance pumping systems, in which asymmetric compression of a compliant tube alters intraluminal transport without requiring a fixed anatomical valve (5). By analogy, sphincter contraction may dynamically impair drainage within an anal fistula tract through functional flow disturbance rather than fixed occlusion.

Based on these considerations, we hypothesized that preservation of anatomical patency alone does not necessarily guarantee effective fistula drainage, and that contraction of the anal sphincter complex may induce a form of dynamic, functional obstruction within the tract.

**FIGURE 1:**
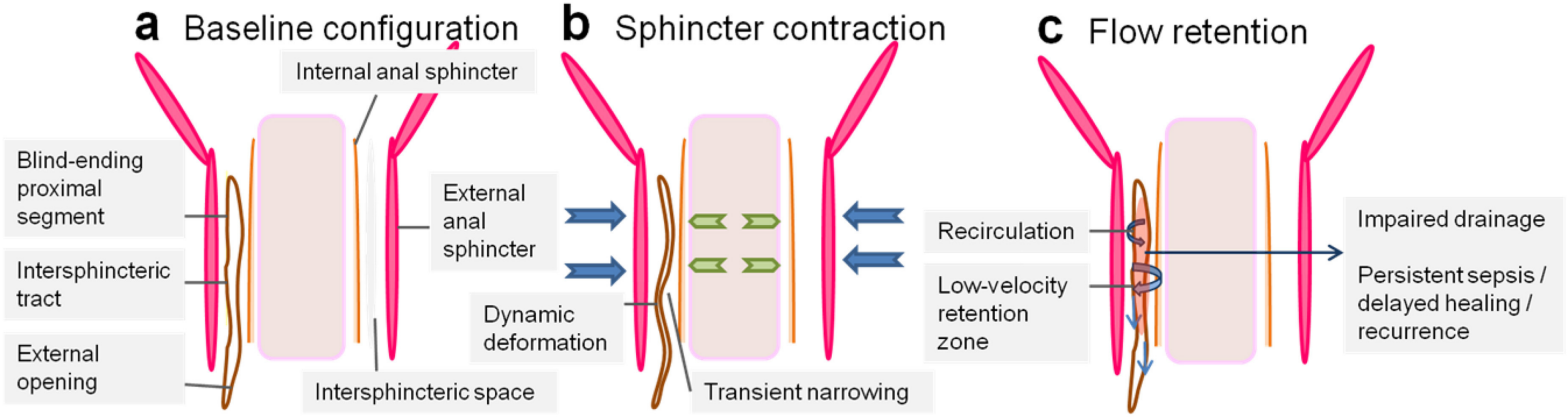
Proposed mechanism of sphincter-induced dynamic obstruction in an intersphincteric anal fistula model. **(a)** Anatomical configuration of a simplified intersphincteric fistula tract located between the internal and external anal sphincters, with a distal external opening at the perianal skin and a blind-ending or retention-prone proximal segment. **(b)** Contraction of the sphincter complex dynamically compresses and deforms the fistula tract, producing transient luminal narrowing despite preserved anatomical patency. **(c)** These dynamic structural changes may disturb intraluminal flow, leading to recirculation, delayed outflow, and low-velocity retention zones that may impair effective drainage. This schematic illustrates the proposed concept of sphincter-induced dynamic obstruction, defined here as a functional, time-dependent impairment of intraluminal drainage rather than a fixed anatomical occlusion.

Figure 1 schematically summarizes this proposed mechanism, linking intersphincteric fistula anatomy, sphincter-induced tract deformation, and the resulting disturbed intraluminal flow patterns. To investigate this hypothesis, we constructed a subject-specific anatomical proof-of-concept fluid–structure interaction model based on pelvic magnetic resonance imaging from a healthy volunteer, into which a synthetic high intersphincteric fistula tract was embedded. We then simulated sphincter contraction and analyzed the spatiotemporal evolution of low-velocity retention zones within the tract. This study was designed as a proof-of-concept computational analysis to test whether sphincter loading can theoretically impair intraluminal flow, rather than to directly validate this mechanism in native fistula patients.

## Materials and methods

### Study subject and ethical approval

One healthy nulliparous female volunteer aged 27 years was prospectively enrolled. She had no history of anorectal surgery, chronic constipation, chronic diarrhea, fecal incontinence, or neuromuscular disorders. The study protocol was approved by the Ethics Committee of China–Japan Friendship Hospital (Approval No. 2021-101-K62), and written informed consent was obtained prior to imaging acquisition.

### MRI acquisition and three-dimensional geometric reconstruction

High-resolution magnetic resonance imaging of the anal canal was performed using a 3.0-T scanner (GE 750). Thin-slice T2-weighted images were acquired with a voxel resolution of approximately 0.75 × 0.75 × 3.0 mm, covering the region from the superior margin of the pubic symphysis to 1.0 cm below the perineum.

DICOM datasets were imported into Mimics (Version 21.0, Materialise, Belgium) for segmentation. The internal anal sphincter, external anal sphincter, and puborectalis muscle were delineated collaboratively by an experienced colorectal surgeon and a radiologist to ensure anatomical accuracy. Three-dimensional reconstruction and surface smoothing were subsequently performed to generate an anatomically consistent solid model of the anal sphincter complex.

### Finite element model and mesh generation

**FIGURE 2:**
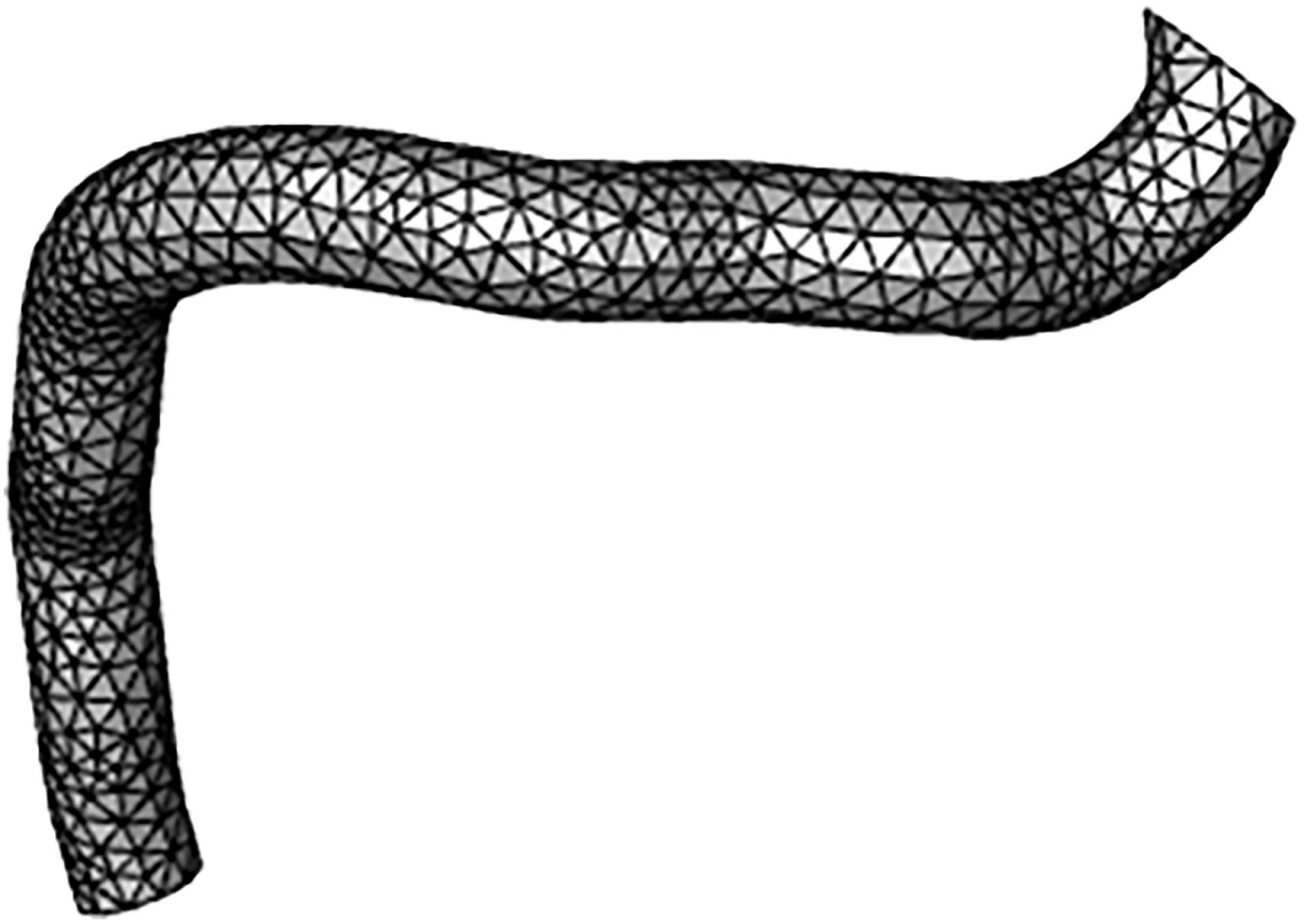
A synthetic high intersphincteric fistula tract was embedded between the internal and external sphincters to create a proof-of-concept fluid–structure interaction model.

The MRI-derived subject-specific anatomical reconstruction of the anal sphincter complex was imported into 3-Matic (Materialise, Belgium) for discretization and mesh generation. A tetrahedral solid-element mesh was applied to all anatomical structures. For modeling simplicity and mechanical continuity, the puborectalis muscle and external anal sphincter were combined into a single structural entity, whereas the internal anal sphincter was modeled separately, the detailed method was shown in previous (8). Because no native fistula was present in the volunteer anatomy, a synthetic curved fistula tract with a diameter of 3 mm was embedded between the internal and external sphincters to represent a typical high intersphincteric configuration (Figure 2). This model should therefore be interpreted as a subject-specific anatomical platform with a synthetic fistula tract for proof-of-concept simulation, rather than as a patient-specific fistula reconstruction. All solid materials were assumed to be linear elastic under small-strain conditions. The elastic modulus of the internal anal sphincter was set to 38.4 MPa based on previously published finite-element modeling data (9). Published biomechanical data for pelvic floor and external anal sphincter tissues remain limited (10, 11). A previous biomechanical study reported a substantially lower modulus for normal anorectal ring muscle tissue (12); however, directly applicable material parameters for chronically inflamed or fibrotic fistula-wall tissue are lacking. The tract modeled in the present study was intended to represent a chronic fistula-related structure located within the intersphincteric plane, where the fistula wall is typically composed of chronically inflamed, granulation, and fibrotic tissue rather than normal muscle alone. Therefore, the mechanical environment of the tract reflects not only the surrounding sphincter complex but also the stiffness contribution of the fibrotic fistula wall and surrounding tissue constraints. In addition, direct use of low-modulus pelvic-floor or sphincter muscle parameters in the present transient bidirectional fluid–structure interaction model may lead to excessive tract deformation and poor mesh stability during sphincter loading. Accordingly, an effective modulus of 10 MPa was used as a model-stabilizing stiffness parameter for the external-sphincter–fistula-wall complex. This value should be interpreted as an effective modeling parameter rather than a patient-specific physiological measurement of normal external sphincter tissue. Mesh convergence testing was performed to ensure numerical stability and solution independence.

### Boundary conditions and loading protocol

The proximal end of the synthetic fistula tract was defined as closed, whereas the distal end was opened to the anal verge and assigned a zero-pressure outlet boundary condition. This closed proximal boundary was selected to represent a simplified blind-ending or retention-prone segment of an intersphincteric infectious tract. In typical cryptoglandular fistula development, infection may spread from the anal gland and intersphincteric space not only distally toward the perianal skin to form an external opening, but also proximally or laterally into adjacent spaces, resulting in blind extensions, residual cavities, or secondary tracts. For this proof-of-concept model, we simplified this complex anatomy into a single intersphincteric tract with a closed proximal end and an open distal outlet, in order to isolate the effect of sphincter-induced deformation on intraluminal drainage. Both ends of the overall model were constrained to maintain mechanical stability and prevent rigid-body motion.

A constant pressure of 9.81 kPa was applied to the luminal surface of the internal anal sphincter to represent baseline tonic contraction. To simulate active sphincter contraction, the external anal sphincter was divided into inferior, middle, and superior layers, each subjected to linearly increasing time-dependent pressure loads. The applied pressures were defined as follows:

Inferior layer: 2.67 + 6.78⋅t kPa

Middle layer: 3.92 + 8.353⋅t kPa

Superior layer: 3.48 + 4.905⋅t kPa

where *t* denotes time in seconds.

A physiological sphincter contraction was simulated over 2 s, producing peak pressures of 16.23 kPa, 20.63 kPa, and 13.29 kPa in the inferior, middle, and superior layers, respectively, to reflect nonuniform contractile behavior.

**FIGURE 3:**
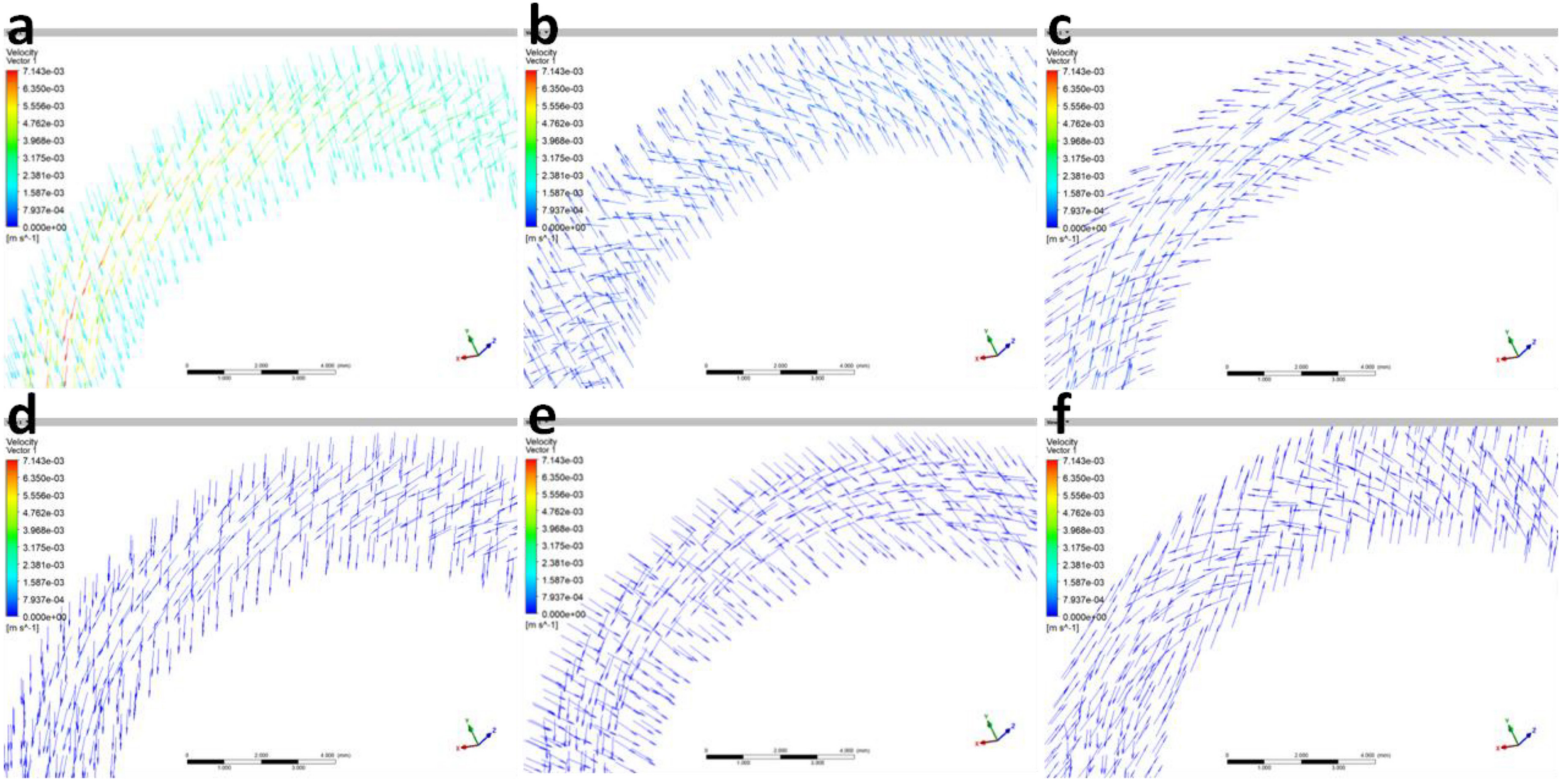
Intraluminal velocity fields during sphincter loading. Pseudocolor velocity vector fields within the fistula lumen at six consecutive time points during simulated anal sphincter loading. **(a–f)** Correspond to 0.01 s, 0.02 s, 0.03 s, 0.04 s, 0.05 s, and 0.06 s, respectively. Velocity magnitude is indicated by the color scale, while arrows denote local flow direction and relative magnitude. Despite anatomical patency of the tract, regions of reduced flow velocity emerge as sphincter loading progresses, providing a qualitative basis for subsequent quantitative analysis of low-velocity retention zones.

The intraluminal velocity field reached a quasi-steady configuration after 0.06 s; therefore, subsequent analyses were performed over 0.01–0.06 s with a time step of 0.01 s (Figure 3).

### Fluid–structure interaction simulation

Bidirectional fluid–structure interaction simulations were performed using ANSYS Workbench to capture the coupled behavior between sphincter deformation and intraluminal fluid flow. The intraluminal fluid was modeled as an incompressible Newtonian purulent exudate-like fluid with a density of 998.3 kg/m^2^ and a dynamic viscosity of 1 Pa⋅s. A laminar flow model was applied, and no turbulence model was used. This setting was used as a simplified approximation of viscous fistula contents. A transient solution scheme incorporating dynamic mesh updating was employed to ensure consistent interaction between the solid and fluid domains. At each time step, deformation of the sphincter complex altered the fistula geometry, which in turn influenced the velocity distribution of the intraluminal fluid.

Pseudocolor velocity maps were exported at each recorded time point for image-based postprocessing and quantitative analysis.

### Image-based inverse mapping pipeline

To quantitatively characterize intraluminal flow behavior in a manner accessible to clinical interpretation, an image-based inverse mapping approach was applied to the velocity pseudocolor maps.

Based on preliminary simulations and the distribution of the velocity color scale, a normalized velocity threshold of 0.4⋅v_max (40% of the maximum velocity) was selected as the primary criterion for defining low-velocity regions. This threshold provided a balance between preserving spatial connectivity of low-velocity zones and minimizing misclassification of high-velocity flow as stagnation.

The colorbar region was extracted from each velocity map, and a linear pixel-to-velocity mapping function was established to convert color information into normalized velocity values (v_i/v_max, range 0–1). Low-velocity masks (v_i < 0.4⋅v_max) were then segmented and subjected to connected-component analysis to quantify the number of fragments and the maximum connected component area.

All image preprocessing and quantitative computations were performed using custom Python scripts to ensure consistent processing across all time frames.

### Quantitative metrics and temporal analysis

To objectively describe the spatiotemporal evolution of intraluminal flow, the following quantitative metrics were extracted from each frame:

Mapping ratio: the proportion of lumen pixels successfully mapped to velocity values, reflecting the effective coverage of inverse mapping.

Low-velocity fraction (low_pct): the proportion of low-velocity pixels relative to mapped lumen pixels under the primary threshold (0.4⋅_max), representing the relative extent of flow stagnation within the mapped lumen area.

Absolute low-velocity fraction (low_frac_total): defined as the product of mapping ratio and low-velocity fraction, representing the proportion of low-velocity pixels relative to the entire image frame and providing a global measure of intraluminal flow stagnation.

Connectivity metrics: including the number of connected components (n_components) and the maximum connected component area (A_max), reflecting fragmentation and aggregation characteristics of low-velocity regions.

Time-series and phase-trajectory analysis: temporal trends of mapping ratio, low-velocity fractions, and connectivity metrics were analyzed, and phase trajectories of A_max versus n_components were constructed to visualize dynamic transitions between aggregation, retention, and fragmentation states.

All analyses were conducted using a unified processing pipeline to ensure comparability across time points.

### Sensitivity analysis

To assess the robustness of low-velocity region identification with respect to threshold selection, a multi-threshold sensitivity analysis was performed. Normalized velocity thresholds of 0.4, 0.5, 0.6, and 0.8⋅v_max were applied to the velocity fields at all six time points (0.01–0.06 s).

For each threshold, the low-velocity area fraction was calculated and summarized in tabular form. Variations in magnitude and temporal trends across thresholds were compared to evaluate sensitivity. Consistent trends and minimal relative variation were interpreted as evidence of robustness and reproducibility of the image-based inverse mapping approach. All sensitivity analyses employed the same masking and mapping pipeline as the primary analysis.

## Results

### Overall temporal trends: coverage and absolute low-velocity fraction

**FIGURE 4:**
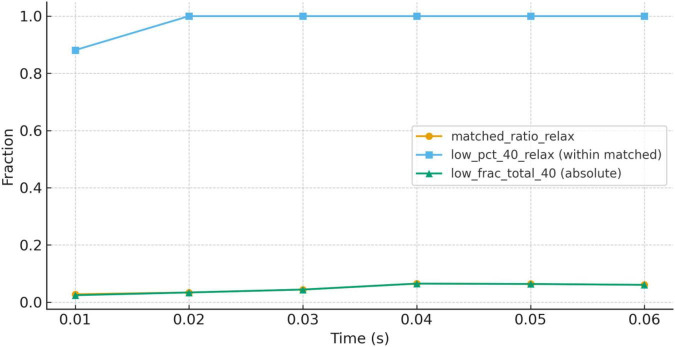
Temporal relationship between mapping coverage of the lumen and low-velocity fraction during sphincter loading. Temporal evolution of the mapping ratio, local low-velocity fraction, and absolute low-velocity fraction within the fistula lumen during simulated sphincter loading. The mapping ratio represents the proportion of the lumen area successfully mapped for velocity analysis at each time point. The local low-velocity fraction denotes the proportion of low-velocity flow within the mapped lumen area, whereas the absolute low-velocity fraction reflects the proportion of low-velocity flow relative to the entire lumen. While the local low-velocity fraction within the mapping region rapidly approached a plateau, the absolute low-velocity fraction increased initially and subsequently stabilized, indicating that the expansion of low-velocity retention zones was not an artifact of regional matching.

Quantitative analysis demonstrated a progressive expansion of low-velocity regions within the fistula lumen during sphincter loading. The mapping ratio increased steadily from approximately 0.027 at 0.01 s to a peak of approximately 0.065 at 0.04 s, indicating a gradual enlargement of the region in which intraluminal velocities could be reliably mapped (Figure 4).

Within the mapped lumen region, the relative low-velocity fraction (low_pct_40) reached 1.00 as early as 0.02 s, suggesting that nearly the entire mapped lumen was dominated by low-velocity flow at this stage. When referenced to the entire image frame, the absolute low-velocity fraction (low_frac_total_40 = low_pct_40 × mapping_ratio) increased from approximately 2.4% at 0.01 s to approximately 6.5% at 0.04 s, followed by a slight decline and stabilization at around 6% during 0.05–0.06 s.

These findings indicate that sphincter contraction rapidly induces widespread low-velocity flow within the fistula lumen. While local stagnation within the mapped region saturates early, the overall burden of low-velocity retention continues to increase and reaches a sustained high level during the mid-loading phase.

### Axial spatial distribution of low-velocity regions

Axial segmentation revealed a heterogeneous spatial distribution of low-velocity regions along the fistula tract (Table 1). The central segment exhibited a marked increase in low-velocity fraction during the mid-loading period, rising from approximately 0.042 at 0.03 s to approximately 0.061 at 0.04 s and further to approximately 0.064 at 0.05 s.

**TABLE 1:** Axial spatial distribution of low-velocity regions within the fistula lumen during sphincter loading.

Frame	Time	total_px	mapped_ px_lumen	mapping_ ratio	low_pct_ 40_relax	low_pct_ 50_relax	low_pct_ 60_relax	mapped_ px_top	mapped_ area_frac_ top	mapped_ px_center	mapped_ area _frac_ center	mapped_ px_bottom	mapped_ area_frac_ bottom
curved_0.01.png	0.01	118,712	3,254	0.027	0.881	0.908	0.911	644	0.027	1,868	0.026	521	0.022
curved_0.02.png	0.02	118,712	3,997	0.034	1	1	1	1,016	0.043	2,015	0.028	598	0.025
curved_0.03.png	0.03	118,712	5,207	0.044	1	1	1	1,523	0.064	3,006	0.042	590	0.025
curved_0.04.png	0.04	118,712	7,678	0.065	1	1	1	1,502	0.063	4,325	0.061	1,232	0.052
curved_0.05.png	0.05	118,712	7,526	0.063	1	1	1	2,182	0.092	4,538	0.064	934	0.039
curved_0.06.png	0.06	118,712	7,160	0.060	1	1	1	1,590	0.067	4,062	0.057	1,175	0.049

Quantitative parameters describing the temporal evolution and axial spatial distribution of low-velocity regions within the fistula lumen during simulated anal sphincter loading. Total pixels indicate the total lumen area, whereas mapped pixels represent the region successfully mapped for velocity analysis at each time point. The mapping ratio is defined as the proportion of mapped lumen pixels relative to the total lumen area. Low-velocity fractions (40%, 50%, and 60% thresholds) denote the proportion of pixels below the corresponding velocity thresholds within the mapped lumen area. Axial subregions (top, center, and bottom) correspond to proximal, middle, and distal segments of the fistula lumen, respectively, and their mapped-area fractions reflect the relative spatial contribution of each segment. The data demonstrate a non-uniform axial distribution of low-velocity regions, with a predominance in the central segment.

In contrast, the proximal and distal segments demonstrated secondary increases in low-velocity fraction during the later phase (0.05–0.06 s). This spatial pattern suggests that low-velocity retention initially emerges in the mid-portion of the fistula—where sphincter-induced compression is most pronounced—and subsequently propagates toward both ends as deformation evolves.

### Connectivity analysis and phase-space trajectory of low-velocity regions

**FIGURE 5:**
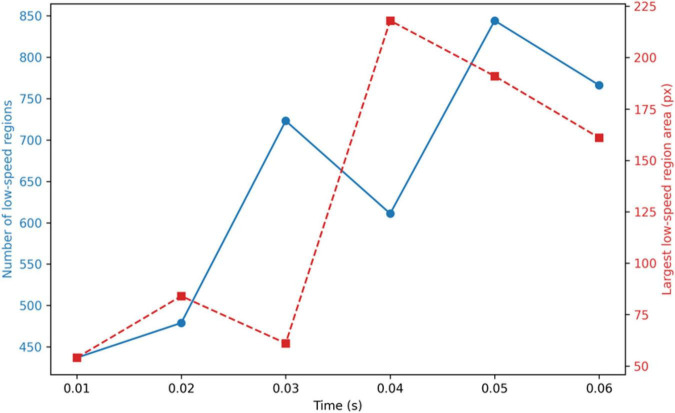
Temporal changes in fragmentation and spatial continuity of low-velocity regions within the fistula lumen. Temporal evolution of the number of discrete low-velocity regions and the area of the largest connected low-velocity region within the fistula lumen during sphincter loading. The number of low-velocity regions reflects the degree of spatial fragmentation, whereas the largest connected region area represents the continuity of low-velocity zones. Variations in these metrics indicate dynamic reorganization of low-velocity regions over time.

Connectivity analysis provided further insight into the dynamic organization of low-velocity regions. The maximum connected component area reached its peak at 0.04 s (218 pixels), whereas the number of connected components peaked later at 0.05 s (844 components) (Figure 5).

**FIGURE 6:**
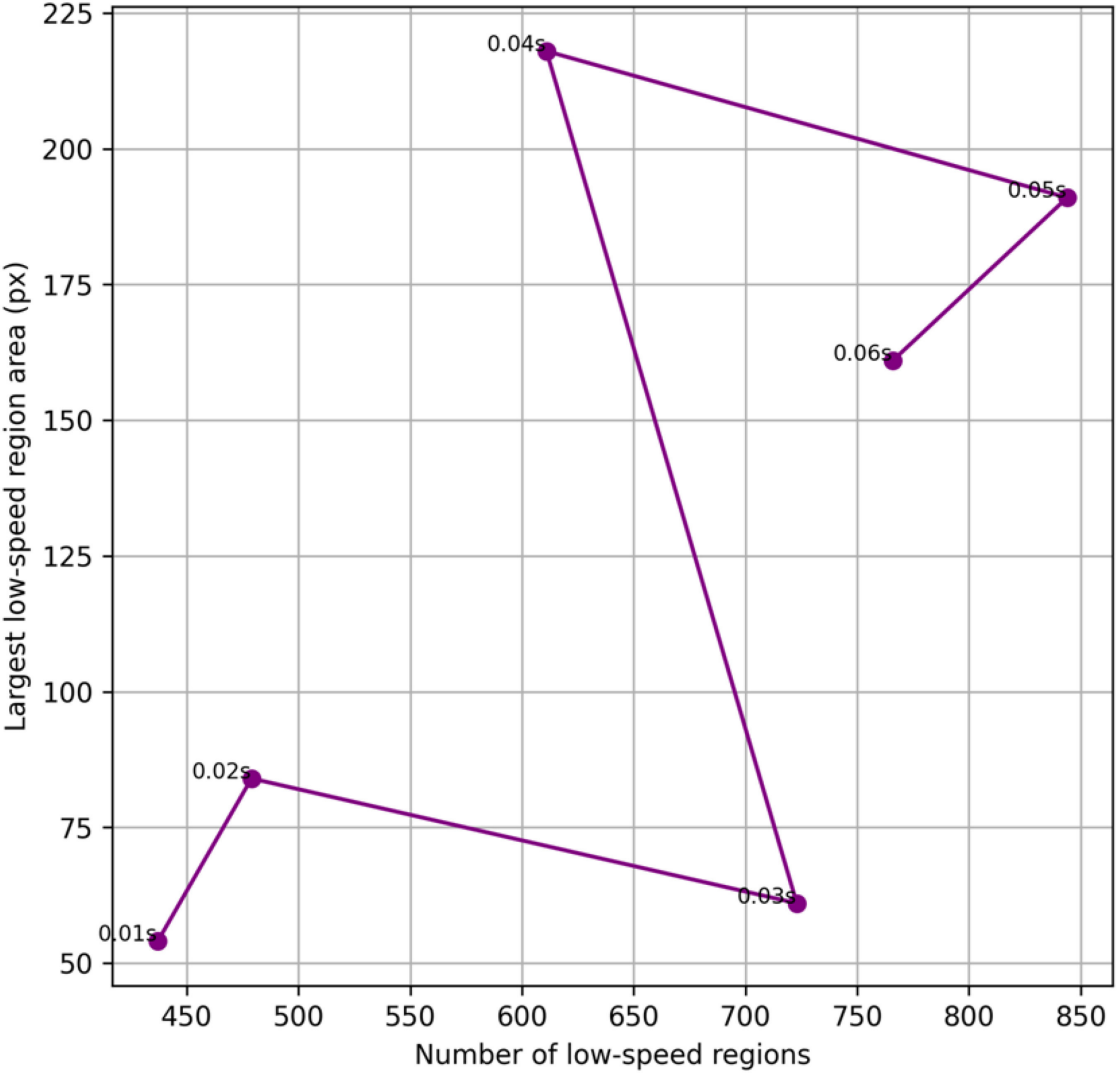
Phase-space trajectory relating fragmentation and spatial continuity of low-velocity regions. The trajectory follows a predominantly counterclockwise pattern, highlighting non-monotonic changes in fragmentation and spatial continuity of low-velocity regions over time.

Phase trajectory analysis of the maximum connected component area versus the number of connected components revealed a counterclockwise loop, indicating a characteristic cyclic evolution of low-velocity regions. Specifically, sphincter contraction first promoted aggregation of low-velocity flow into a dominant core region, followed by a phase of sustained retention, and subsequently by fragmentation into multiple smaller regions while maintaining a high overall low-velocity burden (Figure 6).

This aggregation–retention–fragmentation cycle reflects a dynamic process in which intraluminal flow becomes functionally constrained, partially redistributed, and repeatedly reorganized rather than freely discharged.

### Visual overlays and quantitative annotation

**FIGURE 7:**
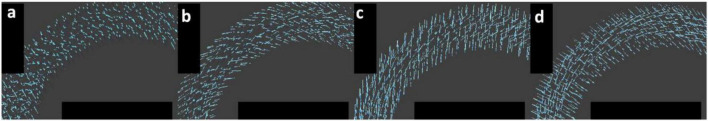
Representative snapshots of low-velocity masks overlaid on intraluminal flow fields. Representative snapshots illustrate low-velocity regions overlaid on intraluminal velocity vector fields of the synthetic fistula tract during sphincter loading. Low-velocity masks were defined according to the prescribed velocity threshold and superimposed on the corresponding flow fields to visualize the spatial distribution and temporal evolution of retention-prone regions. To improve readability, quantitative values that were previously embedded within the panels have been moved to the figure legend. Selected frames highlight characteristic stages of low-velocity region formation, fragmentation, and reorganization observed in the quantitative analyses. **(a)** *T* = 0.02 s, low_pct_40 = 1.000, mapping ratio = 0.034, absolute low-velocity fraction = 0.034. **(b)** *T* = 0.03 s, low_pct_40 = 1.000, mapping ratio = 0.044, absolute low-velocity fraction = 0.044. **(c)** *T* = 0.04 s, low_pct_40 = 1.000, mapping ratio = 0.065, absolute low-velocity fraction = 0.065. **(d)** *T* = 0.06 s, low_pct_40 = 1.000, mapping ratio = 0.060, absolute low-velocity fraction = 0.060.

Representative velocity overlays with superimposed low-velocity masks are shown in Figure 7. To avoid misinterpretation based solely on visual impression, the corresponding mapping ratio and absolute low-velocity fraction for each selected frame are reported in the figure legend. These visual overlays, together with the quantitative values provided in the legend, demonstrate that visually apparent expansion or fragmentation of low-velocity regions is closely aligned with quantitative metrics derived from time-series analysis (Figure 5) and axial segmentation (Table 1). This combined visual–quantitative presentation facilitates intuitive understanding of how sphincter contraction progressively alters intraluminal flow behavior despite persistent anatomical patency.

### Sensitivity Analysis and Robustness of Low-Velocity Identification

Across all tested thresholds (0.4, 0.5, 0.6, and 0.8⋅v_max), threshold-dependent low-velocity fractions showed a consistent relative ordering, indicating that the identification of low-velocity regions was not dominated by a single arbitrary threshold (Table 2). Although the absolute values varied with threshold stringency, the same image-processing pipeline consistently identified low-velocity regions across thresholds. These findings support the robustness of threshold-based low-velocity region identification. The temporal evolution of retention should therefore be interpreted together with the mapping ratio, absolute low-velocity fraction, and connectivity metrics shown in Figures 4–6 and Table 1.

**TABLE 2:** Robustness of low-velocity fraction across multiple velocity thresholds.

Frame	low_pct_40	low_pct_50	low_pct_60	low_pct_80
curved_0.01.png	0.0807	0.0914	0.0985	0.1054
curved_0.02.png	0.0177	0.0183	0.0194	0.0222
curved_0.03.png	0.0055	0.0061	0.0071	0.0101
curved_0.04.png	0.0030	0.0048	0.0068	0.0112
curved_0.05.png	0.00036	0.00171	0.00361	0.00741
curved_0.06.png	0.00040	0.00160	0.00307	0.00724

Low-velocity fractions were calculated using multiple velocity thresholds (0.4, 0.5, 0.6, and 0.8 × vmax) at representative time frames during sphincter loading. The low-velocity fraction in this sensitivity analysis was defined as the proportion of pixels below the specified velocity threshold under the same image-processing pipeline. Although the absolute values varied across thresholds, the relative ordering among thresholds was generally preserved at each time point, indicating that low-velocity region identification was not dependent on a single arbitrary threshold.

## Discussion

The present study demonstrates that contraction of the anal sphincter complex can substantially alter intraluminal flow behavior within a fistula tract through fluid–structure interaction mechanisms. Despite the preservation of anatomical patency, sphincter loading generated extensive low-velocity regions and transient flow retention within the lumen, indicating that effective drainage may be compromised even in the absence of structural obstruction. These findings suggest that fistula drainage should not be considered solely as a function of anatomical openness but also as a dynamic biomechanical process influenced by sphincter activity. Based on this mechanism, we propose the concept of sphincter-induced dynamic obstruction, in which periodic muscular compression leads to transient impairment of fluid transport within the fistula tract. This concept should be understood as a functional, time-dependent impairment of drainage rather than a fixed anatomical occlusion.

Current clinical paradigms emphasize that adequate drainage is the cornerstone of successful management of perianal sepsis and anal fistula. Accordingly, most surgical strategies aim to establish or maintain anatomical patency of the fistula tract, either by creating a wide drainage pathway or by preventing premature closure of the external opening. While this anatomical approach is clinically effective in many cases, it does not fully explain situations in which a fistula tract appears anatomically patent yet continues to exhibit persistent discharge, delayed healing, or recurrence. The findings of the present study provide a potential biomechanical explanation for this discrepancy. Our simulations indicate that periodic sphincter contraction can dynamically modulate fistula geometry and intraluminal flow patterns, generating extensive low-velocity regions that may impede effective fluid transport despite the absence of fixed structural obstruction. In this context, ineffective drainage may arise not only from anatomical blockage but also from transient, mechanically induced impairment of intraluminal flow. A qualitative clinical correlate of this proposed mechanism may be observed on dynamic anorectal ultrasound in patients with anal fistula. In a representative patient, voluntary sphincter contraction was associated with visible bidirectional movement of echogenic intraluminal material within an intersphincteric fistula tract rather than smooth unidirectional evacuation (Supplementary material 1). Although this observation does not provide quantitative validation of the present simulations, it is concordant with the concept that sphincter activity may dynamically disturb intraluminal transport and contribute to transient retention despite anatomical patency. Similar to this concept, clinical and experimental studies on the calf muscle pump have shown that muscle contraction-relaxation cycles can significantly alter venous hemodynamics, including transient blood flow acceleration exceeding the effect of gravity (13, 14). Low-velocity and stagnation-prone flow regions are recognized in multiple biological systems as environments associated with impaired transport efficiency and prolonged fluid residence. In the context of anal fistulas, such regions may reduce effective evacuation of purulent material and inflammatory debris, thereby favoring persistence of infection (15, 16).

This hypothesis should not be interpreted as challenging the cryptoglandular theory or the importance of internal-opening management. We agree that adequate drainage of the septic cavity alone does not necessarily result in closure or obliteration of the internal opening. Rather, our model addresses a complementary issue: residual intersphincteric tracts, blind extensions, or septic cavities may still require effective drainage after inflammatory narrowing, adhesion-related functional closure, or surgical closure of the internal opening. Under these conditions, sphincter-induced dynamic flow impairment may contribute to persistent sepsis, delayed healing, or recurrence by reducing evacuation of purulent material and inflammatory debris. Thus, improved drainage may support local healing but should not be considered a substitute for appropriate treatment of the internal opening.

The present study has several methodological strengths. By integrating subject-specific anatomical reconstruction from magnetic resonance imaging with fluid–structure interaction simulation, the model allowed simultaneous evaluation of sphincter mechanics and intraluminal flow dynamics within a fistula tract. This approach enabled visualization and quantitative analysis of spatiotemporal flow patterns that are difficult to observe using conventional experimental or clinical methods. In addition, the use of image-based velocity mapping and sensitivity analysis across multiple velocity thresholds enhanced the robustness of the observed flow phenomena.

Several modeling assumptions require careful interpretation. First, the synthetic fistula tract was embedded in healthy-volunteer-derived anatomy and therefore did not reproduce the fibrotic, inflamed, or granulation-tissue environment of a native chronic fistula. Such pathological remodeling may substantially alter local stiffness, tract compliance, and deformation under sphincter loading. Second, because the model was based on a single subject, anatomical variability in sphincter thickness, intersphincteric space width, fistula length, curvature, diameter, and proximity to the sphincter complex was not represented and may influence the location and extent of retention zones. Third, the closed proximal boundary was intended to represent a simplified blind-ending or retention-prone segment of an intersphincteric infectious tract, rather than the complete anatomy of all native anal fistulas. In cryptoglandular fistula development, infection may spread distally to form an external opening, while also extending proximally or laterally into adjacent spaces, forming blind extensions, residual cavities, or secondary tracts. However, a widely patent internal opening, communicating abscess cavity, or multiple drainage pathways could alter pressure dissipation and bidirectional exchange, potentially reducing or redistributing flow retention. In addition, the external-sphincter–fistula-wall modulus of 10 MPa should be interpreted as an effective model-stabilizing stiffness parameter rather than a physiological measurement of normal external sphincter tissue. The intraluminal contents were also represented by a simplified Newtonian purulent exudate-like fluid. Finally, because only the contraction/loading phase was simulated, the model cannot determine whether retention persists, dissipates, or reverses during relaxation. These assumptions may affect the absolute magnitude and spatial distribution of the predicted retention zones, and the current results should therefore be interpreted qualitatively rather than as exact quantitative predictions.

## Limitations

Several limitations should also be acknowledged. First, the model was derived from a single healthy volunteer and incorporated a synthetic fistula tract rather than a native patient-specific fistula; therefore, anatomical variability, chronic inflammation, fibrosis, abscess cavities, epithelialized walls, and branching tracts were not fully represented. Second, tissue and fluid properties were approximated using effective modeling parameters rather than direct measurements from patient-specific fistula tissue or discharge. Third, the proximal tract was modeled as closed, representing a simplified retention-prone configuration that may not apply to fistulas with a widely patent internal opening, communicating abscess cavity, or multiple drainage pathways. Fourth, the simulation focused on the contraction/loading phase and did not include a complete contraction–relaxation cycle. Fifth, the dynamic ultrasound observation was included only as a qualitative clinical correlate and does not provide numerical validation of the computational model. Finally, the study evaluated computational flow-related surrogate markers rather than direct clinical outcomes such as discharge volume, bacterial clearance, healing, or recurrence. Accordingly, the findings should be interpreted as qualitative, hypothesis-generating biomechanical evidence and require validation through patient-specific simulations, experimental measurements, and clinical studies.

## Conclusion

In this healthy-volunteer-derived, synthetic intersphincteric fistula model, sphincter contraction generated low-velocity retention zones despite preserved anatomical patency. These findings support a plausible biomechanical hypothesis for ineffective fistula drainage, but should be interpreted as qualitative proof-of-concept evidence pending validation in patient-specific native fistula models and clinical studies.

## Data Availability

The raw data supporting the conclusions of this article will be made available by the authors, without undue reservation.
