# Supplementary material for: Sphincter-induced dynamic obstruction in a synthetic intersphincteric anal fistula model: a proof-of-concept fluid–structure interaction study
Source: Front Med (Lausanne). 2026 Jul 10;13:1865558. doi: 10.3389/fmed.2026.1865558 (PMC13395717; doi:10.3389/fmed.2026.1865558)

**Supplementary Video legend**

**Supplementary Video S1.** Endoanal ultrasonography showing bidirectional movement of purulent content within the fistula tract during anal sphincter contraction and relaxation. From 2 to 6 s, sphincter contraction is associated with upward flow in the image, indicating extrusion out of the fistula tract. From 6 to 8 s, sphincter relaxation is associated with downward reflux in the image, indicating suction back into the fistula tract. After 8 s, the sphincter returns to the resting state without obvious directional flow. The white arrow indicates upward flow out of the fistula tract, and the black arrow indicates downward reflux back into the fistula tract.

**Supplementary Figure legend**

**Supplementary Figure S1.** Representative endoanal ultrasound image showing bidirectional movement of purulent content within the fistula tract. The highlighted region indicates the fluid-containing portion of the tract. The white arrow indicates upward flow in the image, corresponding to extrusion out of the fistula tract during sphincter contraction, and the black arrow indicates downward reflux in the image, corresponding to suction back into the fistula tract during sphincter relaxation.


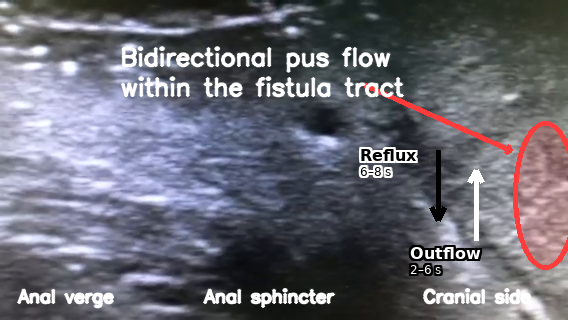

Supplement: Supplementary file 1 [file Table_1.docx]
